# Dielectric and ferroelectric sensing based on molecular recognition in Cu(1,10-phenlothroline)_2_SeO_4_·(diol) systems

**DOI:** 10.1038/ncomms14551

**Published:** 2017-02-20

**Authors:** Heng-Yun Ye, Wei-Qiang Liao, Qionghua Zhou, Yi Zhang, Jinlan Wang, Yu-Meng You, Jin-Yun Wang, Zhong-Ning Chen, Peng-Fei Li, Da-Wei Fu, Songping D. Huang, Ren-Gen Xiong

**Affiliations:** 1 Ordered Matter Science Research Center, Southeast University, JiuLongHu campus, JiangNing, Nanjing 211189, China; 2 Department of Physics, Southeast University, Nanjing 211189, China; 3 Fujian Institute of Research on the Structure of Matter, The Chinese Academy of Sciences, Fuzhou 350002, China; 4 Department of Chemistry and Biochemistry, Kent State University, Kent, Ohio 44240, USA

## Abstract

The process of molecular recognition is the assembly of two or more molecules through weak interactions. Information in the process of molecular recognition can be transmitted to us via physical signals, which may find applications in sensing and switching. The conventional signals are mainly limited to light signal. Here, we describe the recognition of diols with Cu(1,10-phenlothroline)_2_SeO_4_ and the transduction of discrete recognition events into dielectric and/or ferroelectric signals. We observe that systems of Cu(1,10-phenlothroline)_2_SeO_4_·(diol) exhibit significant dielectric and/or ferroelectric dependence on different diol molecules. The compounds including ethane-1,2-diol or propane-1,2-diol just show small temperature-dependent dielectric anomalies and no reversible polarization, while the compound including ethane-1,3-diol shows giant temperature-dependent dielectric anomalies as well as ferroelectric reversible spontaneous polarization. This finding shows that dielectricity and/or ferroelectricity has the potential to be used for signalling molecular recognition.

## Abstract (web summary)

Molecular recognition is an important biological process where guest and host molecules interact through non-covalent bonding. Ye *et al*. show that this can be sensed by the dielectric and ferroelectric signals of the final complexes in a series of metal-coordination compounds with different diol molecules.

Molecular recognition is the weak binding of a guest molecule to a complementary host molecule to form a host–guest complex through non-covalent bonding interactions such as hydrogen bonding, π–π stacking, metal coordination, hydrophobic forces and cation–π interaction^1^,^2^,^3^,^4^,^5^,^6^. Molecular recognition plays important roles in many biological processes, ranging from enzymatic catalysis, protein synthesis to immunity^7^,^8^. Recently, much attention has been directed to the artificial materials and devices based on molecular recognition and related supramolecular chemistry^9^,^10^,^11^,^12^,^13^. These host–guest systems can achieve specific functions, such as molecular machines, molecular imprinting, switching, sensing and separation^14^,^15^,^16^. One of the characteristic features of the host–guest systems is that the guest molecule has large freedom of motion because of the weak binding interaction and spacious room for molecular motion. In general, the dynamical state of the guest molecule depends on the internal structural parameters and external environment such as temperature. Physical quantities sensitive to the state of the motion, such as alternating current (ac) dielectric constant, can be utilized for signalling in the process of molecular recognition.

In the past few years, host–guest systems have been found to be intrinsically related to rich dielectric and ferroelectric properties. The temperature-induced dynamic changes of the dipolar guest molecules can lead to significant dielectric responses and symmetry breaking at particular temperatures in some cases. Several systems, such as metal formate frameworks^17^,^18^,^19^, organic ammonium-crown inclusion compounds^20^,^21^,^22^,^23^ and other coumpounds^24^,^25^, have been investigated. However, no systematic work has been carried out to study how the dielectric and ferroelectric properties sense different guest molecules. We herein describe dielectric and ferroelectric properties of new systems that include different diol molecules. We found that these systems show significant dependence of dielectric and ferroelectric properties on different included molecules. This finding shows that the dielectric and ferroelectric signals might be used for sensing in molecular recognition.

## Results

### Structural phase transition

**Figure 1:**
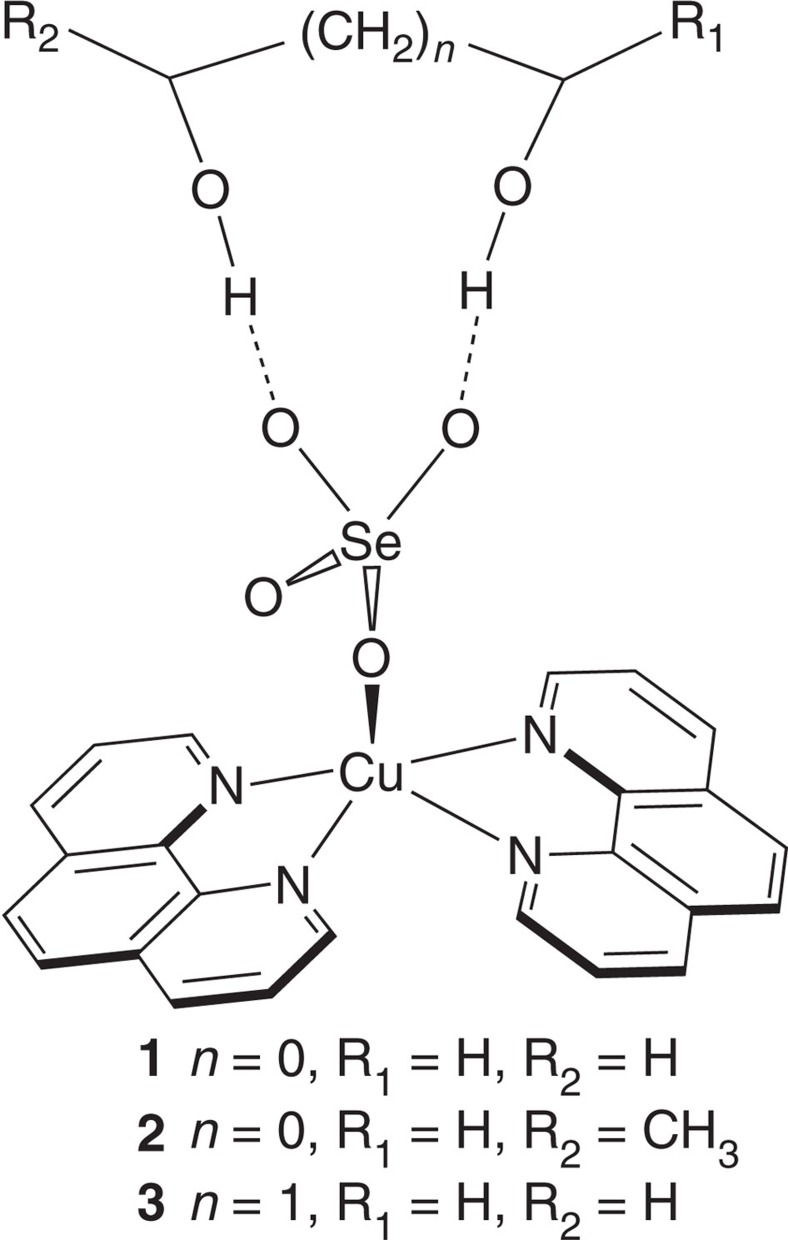
Structural formula of compounds 1–3. The dashed lines indicate hydrogen bonding interactions.

**Figure 2:**
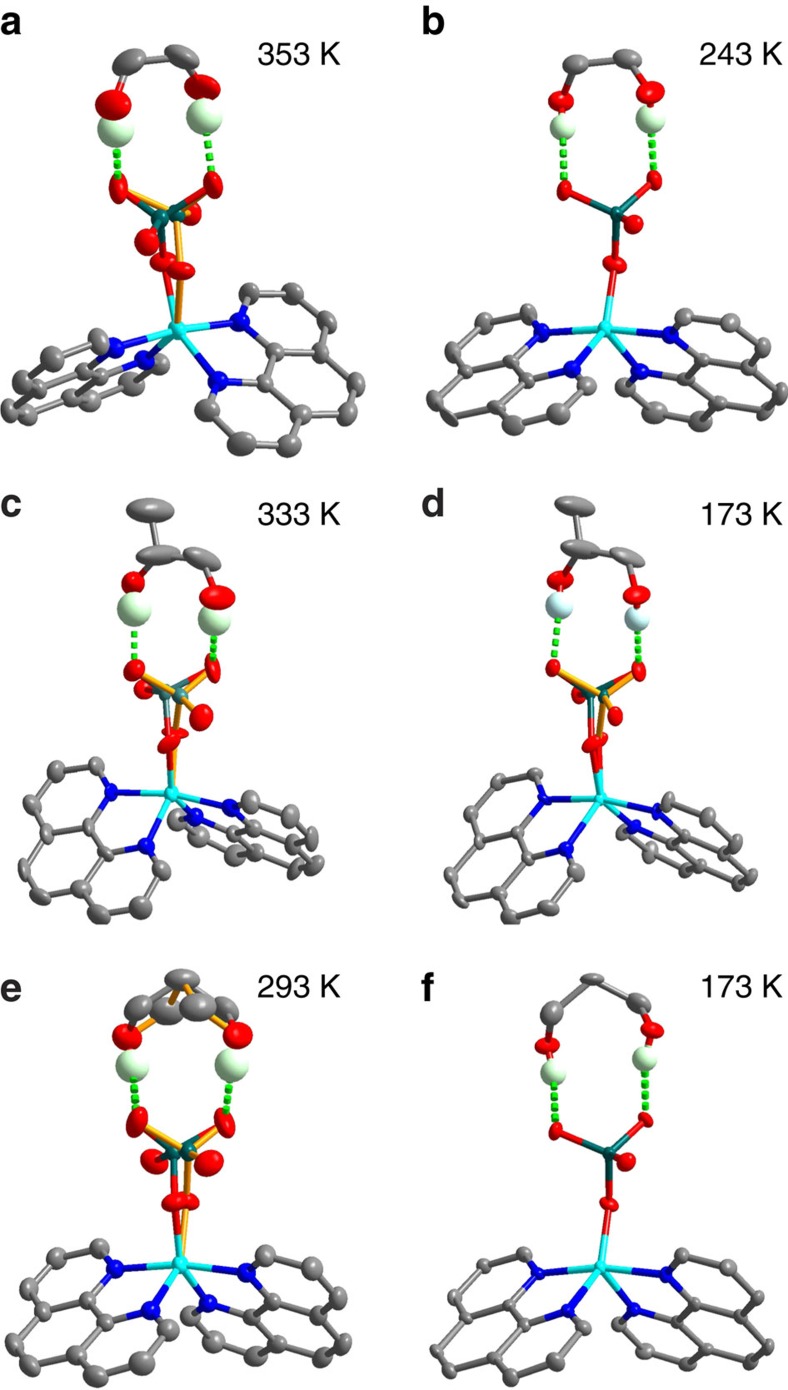
Molecular structures of 1–3. (**a**,**b**) Molecular structures of **1** in the HTP and LTP, respectively. (**c**,**d**) Molecular structures of **2** in the HTP and LTP, respectively. The ratios of the two orientations of the SeO_4_^2−^ anion are 0.53:0.47 and 0.88:0.12, respectively. (**e**,**f**) Molecular structures of **3** in the HTP and LTP, respectively. The temperatures indicate those at which the structures were determined, respectively. The green dashed lines indicate hydrogen bonding interactions. The two orientations of the disordered SeO_4_^2−^ anion were distinguished by the two-coloured and the orange bonds. H atoms bonded to the C atoms were omitted for clarity.

Solvate compounds Cu(1,10-phenothroline)_2_SeO_4_·(diol) (Fig. 1) were obtained as crystals by recrystallization of Cu(1,10-phenothroline)_2_SeO_4_ from diols. They were found to undergo temperature-triggered structural phase transitions by thermal analysis (Supplementary Fig. 1) and dielectric measurements. We determined the temperature-variable crystal structures by X-ray diffraction to understand the origins of the phase transitions and mechanisms of their molecular recognition. These compounds consist of monomeric complex Cu(1,10-phenanthroline)_2_SeO_4_ and diol molecules. The central Cu^2+^ ion has a distorted square-pyramidal coordination geometry defined by four N atoms from two chelating 1,10-phenanthroline ligands and one O atom from a monodentate SeO_4_^2−^ anion, and the apex is occupied by a N atom. The monomeric complex and diol molecules are held together by O-H⋯O hydrogen bonding interactions, giving supramolecular structures with an 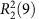 ring motif for Cu(1,10-phenothroline)_2_SeO_4_·(ethane-1,2-diol) (**1**) and Cu(1,10-phenothroline)_2_SeO_4_·(propane-1,2-diol) (**2**) and an 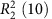 ring motif for Cu(1,10-phenothroline)_2_SeO_4_·(propane-1,3-diol) (**3**) (Fig. 2).

**Figure 3:**
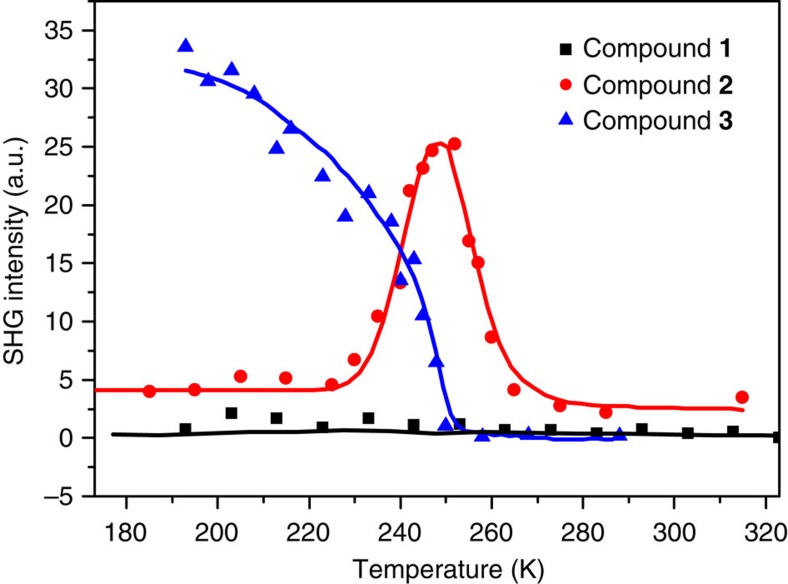
SHG responses of compounds 1–3. Solid lines are a guide to the eye.

The phase transition temperature of **1** is around 325 K in the cooling run. The high-temperature phase (HTP) structure at 353 K has the centrosymmetric space group *C*2/*c* (for crystallographic information, see Supplementary Table 1 and Supplementary Data 1–3). The supramolecule of Cu(1,10-phenothroline)_2_SeO_4_·(ethane-1,2-diol) is located on the crystallographic *C*_2_ axis passing through the Cu atom and the centre of the C-C bond of the ethane-1,2-diol molecule. The SeO_4_^2−^ anion adopts two-fold orientational disorder to satisfy the symmetry requirement, and two O atoms, which are not involved in the hydrogen bonds, distribute over two positions, respectively. The low-temperature phase (LTP) structure at 243 K has the space group *P*2_1_/*c*, with the *b* axis tripling with respect to that of the HTP. The coordination geometry of the Cu atom and the molecular geometry are comparable to those in the HTP. The main difference between the LTP and HTP structure is the ordering of the SeO_4_^2−^ anion, and thus the phase transition can be understood as driven by the ordering of the SeO_4_^2−^ anion. To identify the symmetry, we employed second harmonic generation (SHG) spectroscopy, which is an optical method effectively identifying non-centrosymmetric structures^26^,^27^,^28^. As shown in Fig. 3, there is no SHG response observed on **1**, which is consistent with the centrosymmetric space groups.

The phase transition temperature of **2** is around 300 K in the cooling run. All structures at 333, 298, 253, 243, 173 and 93 K were refined in the space group *Cc* (for crystallographic information, see Supplementary Table 2 and Supplementary Data 4–9). Their difference is in the population of the two orientations of the SeO_4_^2−^ anion. The ratio of the two orientations changes from 0.53:47 to 0.90:0.10 as the temperature decreases from 333 to 93 K. In Fig. 3, a finite SHG response was observed on **2** in the whole measured temperature range with an anomaly, supporting the symmetry assignment in the structural refinements. Compared with the HTP structure of **1**, the structures of **2** do not possess the *C*_2_ symmetry, due to: (1) the propane-1,2-diol molecule lacks the *C*_2_ symmetry; (2) the ratios of the two orientations of the SeO_4_^2−^ anion deviate from 0.5:0.5. Such a structure model has been adopted for the sulfate analogue^29^.

**Figure 4:**
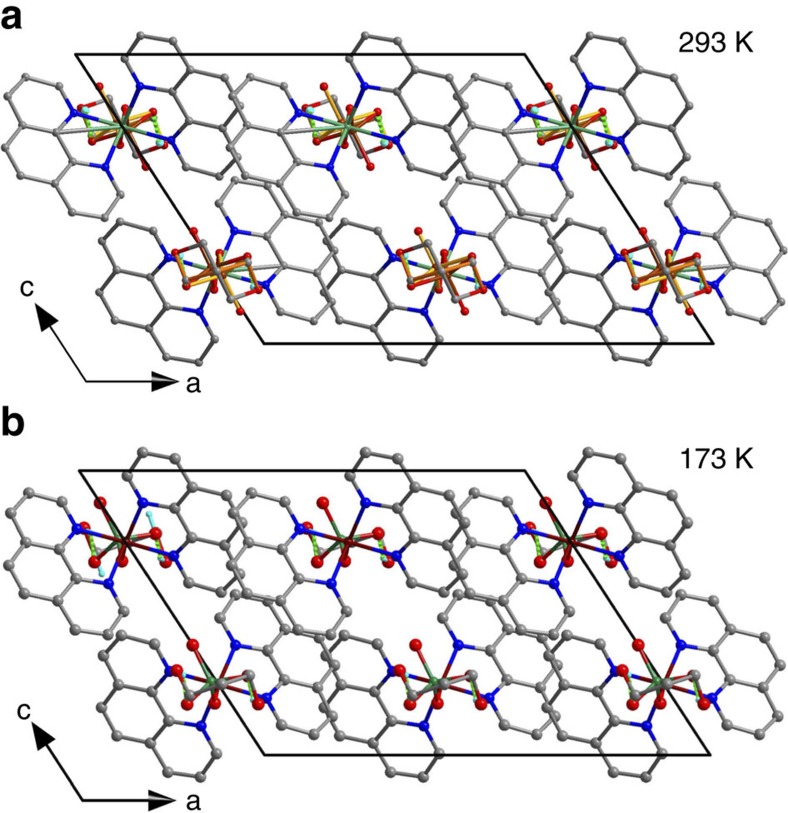
Comparison of packing diagrams of 3 in the HTP and LTP. The comparison reveals the similarities of the lattices and the differences in the orientational states of the SeO_4_^2−^ anions and the propane-1,3-diol molecules. (**a**) Projection along the common *b* axis at 293 K. (**b**) Projection along the common *b* axis at 173 K.

Compound **3** undergoes a phase transition at around *T*_c_=260 K. The HTP structure at 293 K has the centrosymmetric space group *C*2/*c* (for crystallographic information, see Supplementary Table 3 and Supplementary Data 10 and 11). The supramolecule is located on the crystallographic *C*_2_ axis, and the SeO_4_^2−^ anion and propane-1,3-diol molecule are disordered over two orientations with the equal populations, respectively. Each orientation of the propane-1,3-diol has the intermolecular *C*_2_ axis superimposed with the crystallographic *C*_2_ axis. The LTP structure at 173 K assumes the polar space group *Cc*. Both the SeO_4_^2−^ anion and propane-1,3-diol molecule become ordered. Obviously, the ordering leads to the *C*_2_-symmetry-breaking phase transition (Fig. 4). The symmetry transition was confirmed by SHG measurements. As shown in Fig. 3, the occurrence of SHG signal at around *T*_c_ in the cooling run indicates a transition from centrosymmetry to non-centrosymmetry. According to the symmetry change, the crystal belongs to 2/*m*F*m* species of the 88 kinds of ferroelectrics^30^.

### Dielectric and ferroelectric properties

**Figure 5:**
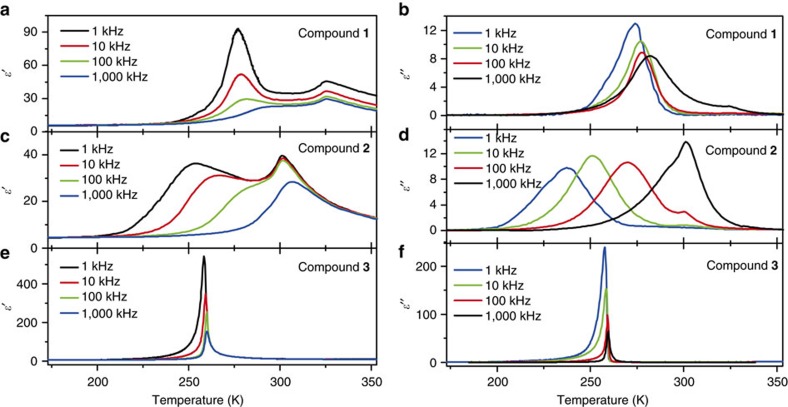
Dielectric responses of **1**–**3**. (**a**,**c**,**e**) Temperature dependences of the real part *ɛ*′ of complex dielectric constant measured along the *a* axis at different frequencies for **1**–**3**. (**b**,**d**,**f**) Temperature dependences of the imaginary part *ɛ*″ of complex dielectric constant for **1**–**3**.

**Figure 6:**
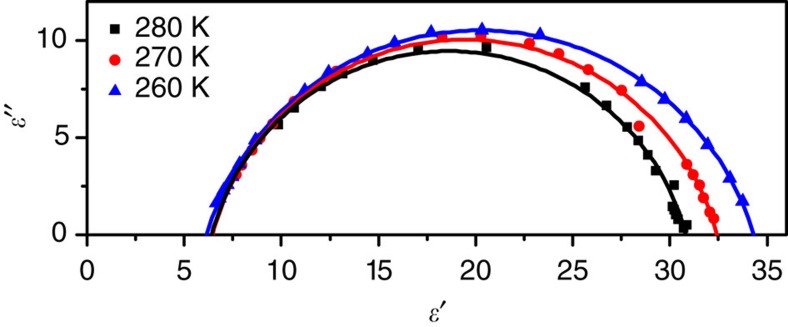
Argand plots of the complex dielectric constant of 2. The dielectric complex dielectric constants in the temperature range of the dielectric anomalies were used for the plots. The solid lines represent the best fits using the Cole–Cole model.

The structural analysis reveals the slowing down of dynamics of the SeO_4_^2−^ anion with decreasing temperature in **1**–**3**. Such a process is usually accompanied by a dielectric response, which possibly contributes to transmitting the signal of molecular recognition. We measured temperature-variable complex dielectric constant (*ɛ*) (*ɛ*=*ɛ*′−*iɛ*″, where *ɛ*′ is the real part and *ɛ*″ is the imaginary part) for single crystal samples of the three compounds. As shown in Fig. 5, **1** and **2** show the similar dielectric response, while **3** exhibits distinct dielectric behaviour. For **1** and **2**, two anomalous peaks with a temperature gap of about 30–50 K at each measured frequency were observed. It is natural to associate the two anomalies with two structural phase transitions. However, the heat capacity measurements just show one wide thermal anomaly, indicating a single phase transition (Supplementary Fig. 1). The crystal structures determined at intermediate temperatures (303 and 243 K for **1** and **2**, respectively) have the same space group as those of the HTPs. Probably, two different polar mechanisms are responsible for the two sequential dielectric anomalies. The anomaly at higher temperature is mild in comparison of those in ferroelectric phase transitions^31^ or in transitions involving rotational dipoles^32^,^33^, and does not show significant frequency dependence. Such a dielectric response is usually due to the electronic and ionic polarization. The anomaly at lower temperature shows strong frequency dependence in both real and imaginary part (Supplementary Fig. 2). Take **1** as example, the peak value of *ɛ*′ decreases from 95 to 27 and the peaking temperature moves from 280 to 295 K as the frequency increases from 1 kHz to 1 MHz. The low-frequency dispersion is attributable to the dielectric relaxation due to the reorientation of dipoles (SeO_4_^2−^ anion). To analyse the relaxation process, the complex dielectric constant of **2** is plotted in Argand diagram and fitted by the Cole–Cole model with the following function^34^:


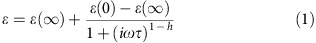


where *ɛ*(0) and *ɛ*(∞) are the low-frequency and high-frequency values of the real part of dielectric constant, *τ* is the relaxation time, *ω* is the angular frequency and *h* is a measure of the distribution of relaxation time. As shown in Fig. 6, the data from ‘Cole–Cole arcs' with their centres located below the *ɛ*′ axis indicate a polydispersive character. The fitted *h* values are 0.1835, 0.1628 and 0.1612 at 260, 270 and 280 K, respectively, and *τ* values are 5.3 × 10^−6^, 1.8 × 10^−6^ and 6.37 × 10^−7^ s, respectively. A good fit of Cole–Cole model supports that the relaxation process is the reorientation of dipoles, and the low *h* value indicates a narrow distribution of the relaxation time. The same analysis was also carried out for **1**. The curves deviate significantly from Cole–Cole arcs, indicating a more complex dielectric relaxation process in **1**.

For **3**, only one *λ*-shape anomalous peak appears at each frequency, and the peak heights are significantly larger than those for **1** and **2** in orders of magnitude. The large dielectric constant anomalies reveal the ferroelectric nature of the transition. In the vicinity of the critical temperature, the dielectric response shows Curie–Weiss behaviour, *ɛ*′=*C*_p_/(*T*−*T*_0_) (*T*>*T*_c_) or *C*_f_/(*T*_0_′−*T*) (*T*<*T*_c_). The fitted Curie constants at 100 kHz is *C*_p_=322 K and *C*_f_=154 K, and Weiss temperatures *T*_0_≈*T*_0_′=259.3 K. The *C*_p_/*C*_f_ ratio of 2.09 is quite close to the theoretical value (*C*_p_/*C*_f_=2) expected for a second-order ferroelectric phase transition. The fitted Curie constants at other frequencies are included in Supplementary Table 4.

**Figure 7:**
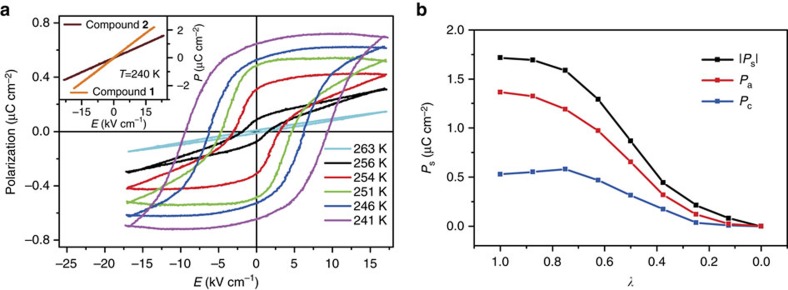
Properties of polarization switching for 3. (**a**) *P*−*E* hysteresis loops measured at different temperatures along the *a* axis by the Sawyer–Tower circuit method. Inset: *P*−*E* dependences of **1** and **2** at 240 K. (**b**) Calculated ferroelectric polarization along the path connecting the centrosymmetric (*λ*=0) to polar structure (*λ*=1). Both the module (black) and components of the vector along the *a* and *c* axis (red/blue) were plotted.

To identify ferroelectricity, observation of polarization–electric field (*P*−*E*) hysteresis loops using the Sawyer–Tower circuit is a reliable method^31^. Thus, we examined the *P*−*E* dependence of the three compounds (Fig. 7). Compounds **1** and **2** just show the linear dependence at various temperatures, indicating no switchable spontaneous polarization and the lack of ferroelectricity (inset of Fig. 7a). For **3**, the polarization response is also linear at temperature above *T*_c_, as expected for a paraelectric phase. At a temperature close to *T*_c_ (256 K), a flat loop was observed, and a non-zero remnant polarization (*P*_r_) at zero field appeared, corresponding to a transition state. Perfect loops were developed at lower temperatures in the stable ferroelectric phase. At 241 K and 50 Hz, we obtained *P*_s_=0.70 μC cm^−2^, *P*_r_=0.65 μC cm^−2^ and coercive field (the intercept of the loop with the field axis) *E*_c_=7.1 kV cm^−1^. Compared with those in other recently developed molecular ferroelectrics^17^,^18^,^22^,^23^,^27^,^28^,^35^,^36^,^37^,^38^,^39^,^40^,^41^,^42^,^43^, *P*_s_ of **3** at 241 K is among the moderate level.

## Discussion

**Figure 8:**
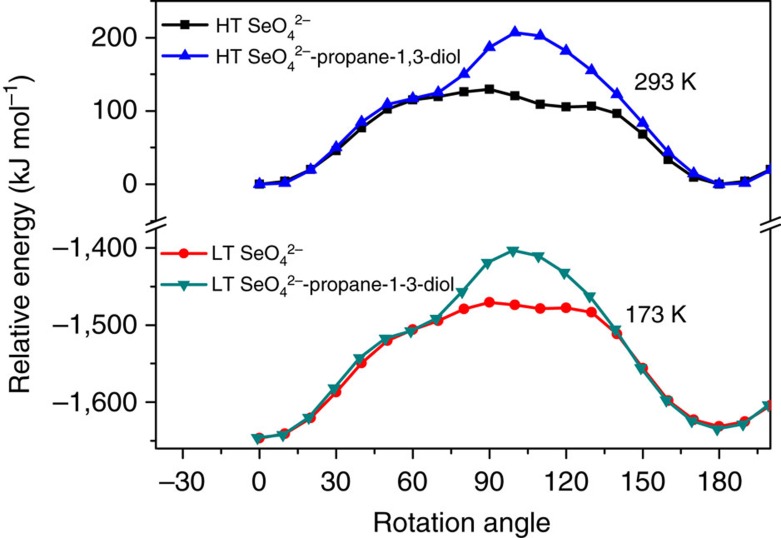
Energy barrier of molecular rotation in **3**. The relative energies are calculated with the rotation angles from 0° to 200° for the rotation types A and B in both the HTP and LTP. The energy barrier difference for the two rotation types is about 80 kJ mol^−1^.

The ferroelectric mechanism can be interpreted by a combination of structural analysis and theoretical calculation. For compound **3**, the molecular electronic dipole moment can be taken as pointing from the Se to Cu atom, since the positive and negative charges are carried mainly by Cu^2+^ ion and SeO_4_^2−^ anion, respectively. In the paraelectric phase, the two orientations of SeO_4_^2−^ distribute over the *C*_2_ axis in the [0 1 0] direction, and the dipoles in the *ac* plane are antiparallel and cancel each other. In the ferroelectric phase, the SeO_4_^2−^ anion becomes ordered with a single orientation. The supramolecules in the *Cc* space group are related by the translations or glides, and thus the dipoles in the *ac* plane are arranged in parallel, leading to the occurrence of spontaneous polarization (Supplementary Fig. 3). Since the spontaneous polarization in **3** originates from the loss of the *C*_2_ axis, the path of polarization reversal can be assumed as rotation of SeO_4_^2−^ anion (type A) or SeO_4_^2−^-diol as a rigid part (type B) around the (pseudo) *C*_2_ axis (Supplementary Fig. 4). To figure out the detail, we calculated energy barriers for the two rotation types, as shown in Fig. 8. The energy barrier difference of about 80 kJ mol^−1^ indicates that type A is more favourable in **3**. This calculation also reveals that the centrosymmetric structure is higher in energy than the ferroelectric one, as expected. Beside these approaches, other possible contributions to the polarization, such as intramolecular charge transfer, are negligible (Supplementary Fig. 5 and Supplementary Table 5).

With this mechanism, we evaluated the crystal polarization by the Berry phase method using a periodic unit cell. The calculated polarization vector of the LTP lies in the *ac* plane perpendicular to the *C*_2_ axis. The vector module is 1.71 μC cm^−2^ and its component along the *a*-direction is 1.36 μC cm^−2^, which reproduces the experimental value of 0.7 μC cm^−2^. The continuous evolution of polarization (both the module and components in the *a*/*c*-direction) from the centrosymmetric (*λ*=0) to the polar structure (*λ*=1) was plotted as a function of dimensionless parameter *λ* in Fig. 7b. The dimensionless parameter *λ* is the normalized amplitude of the atomic displacements in the path from the centrosymmetric structure (*λ*=0) to the polar structure (*λ*=1). Both the rotation of SeO_4_^2−^ anion and slight displacement of other atoms are implied in *λ*.

The primary feature distinguishing ferroelectrics from other pyroelectrics is that ferroelectric spontaneous polarization can be reversed with an applied electric field^31^. Ferroelectric spontaneous polarization is generated by symmetry breaking, and correspondingly, the crystal structures with the opposite orientation of the polarization are identical or enantiomorphous, and can be transformed into each other by the symmetry operation which is kept just in the paraelectric phase. The two polarization states in **3**, for instance, are related by the *C*_2_ symmetry. For **2**, the dipoles in both the HTP and LTP should be arranged in the same manner as in **3**. The ferroelectric polarization reversal requires type B rotation, or the two polarization states will be not symmetrically equivalent, because propane-1,2-diol molecule lacks the *C*_2_ symmetry. However, the barrier energy of rotation type B in **2** is 45 kJ mol^−1^ higher than that of rotation type A (Supplementary Fig. 6), indicating that type B rotation is unfavourable and ferroelectric polarization reversal is impossible in the investigated temperature range. The only isosymmetric phase transition in **2** also suggests the difficulty in the polarization reversal. Such polar compounds like **2** are usually regarded as pyroelectrics.

As for **1,** the phase transition may involve the type B rotation since the energy barriers for two rotation types are almost equal (Supplementary Fig. 6), different from those in **2** and **3**. Although it also undergoes a *C*_2_-symmetry-breaking transition, the LTP remains centrosymmetric, and the two orientations of the SeO_4_^2−^ anion retain in the crystal with the equal population (Supplementary Fig. 7). Therefore, **1** has no ferroelectric spontaneous polarization.

In summary, diols are recognized by Cu(1,10-phenlothroline)_2_SeO_4_ through hydrogen bonding interactions to form crystalline compounds with a general formula Cu(1,10-phenlothroline)_2_SeO_4_·(diol). These compounds exhibit distinct dielectric and/or polar behaviours, depending on the included diol molecules. Both the HTP and LTP of **1** are centrosymmetric, and thus the crystal shows no polarization. Both the HTP and LTP of **2** are polar, and thus the crystal shows non-switchable polarization. Their phase transitions are accompanied by the moderate dielectric response. Compound **3** has the centrosymmetric HTP and the polar LTP, and thus shows switchable polarization (ferroelectricity) and giant dielectric response. Since the dielectric/ferroelectric properties show high dependence on included diol molecules, the present crystalline compounds offer very attractive perspectives as models of dielectric/ferroelectric sensing. The finding will throw light on the further research on the dielectric/ferroelectric sensing based on molecular recognition, and thus expand the application of molecular ferroelectric materials. From the view point of molecular design, the models can be easily extended to other systems, because the bidentate ligand, diol molecule and the metal ion can be tuned in a wide of range. It is expected that the selenate group can be easily replaced by the sulfate group to maintain the similar structures. Research on the dielectric/ferroelectric sensing properties of these inclusion compounds is in progress.

## Methods

### Synthesis

1,10-Phenanthroline (10.0 mmol, 1.80 g), copper(II) carbonate basic (2.5 mmol, 0.55 g) and selenic acid (40%, 2.00 g) were placed in a 500 ml flask with distilled water (5 ml) and ethane-1,2-diol (200 ml) as solvents. After refluxing for 4 h at 393 K, the solution was cooled to room temperature and then filtered into a 250 ml beaker. Green block crystals of **1** were obtained by slow evaporation of the filtrate at 373 K. Green block crystals of **2** and **3** were prepared using a similar method by replacing ethane-1,2-diol with propane-1,2-diol and propane-1,3-diol, respectively. The purity of the bulk phases was verified by X-ray powder diffraction, infrared and UV–vis spectra (Supplementary Figs 8–10).

### Experimental characterization

Methods of DSC, SHG, dielectric, pyroelectric and *P*−*E* hysteresis loop measurements were described elsewhere^22^,^44^. For dielectric, *P*−*E* hysteresis loop measurements, single-crystal plates with about 5 mm^2^ in area and 0.5 mm in thickness were cut from the large crystals in the [1 0 0] direction. Silver conduction paste deposited on the plate surfaces was used as the electrodes.

### Computational details

The crystalline property calculations were performed within the framework of density functional theory (DFT) implemented in the Vienna *ab initio* Simulation Package. The exchange–correlation interactions were treated within the generalized gradient approximation of the Perdew–Burke–Ernzerhof type. The spontaneous polarization was evaluated by the Berry phase method developed by King-Smith and Vanderbilt. A unit cell with period boundary conditions was used to simulate the bulk crystal. The initial model for the calculation of the polarization was derived from the crystal structure of the ferroelectric phase (*λ*=1). The models of the transition states were obtained by the clockwise or counter-clockwise rotation of half of the SeO_4_^2−^ anions around the (pseudo) *C*_2_ axis. For each transition state (0<*λ*<1), we took an average of polarization calculated from the models by the clockwise or counter-clockwise rotation (Supplementary Fig. 11).

Energy barrier calculations were carried out with the Gaussian 09 software package. The model structures considering the effects from neighbouring molecules were extracted from the X-ray crystallographically determined geometries (Supplementary Fig. 4). The total energy calculations of structures at different temperatures and rotation angles (*R*) around the (pseudo) *C*_2_ axis were performed by the DFT method, B3LYP-D3, with the Grimme's DFT-D dispersion correction term, in combination with Stuttgart–Dresden–Bonn relativistic effective core potential SDD used for the Cu atoms (which replaces 10 electrons in inner shells 1 and 2, leaving 17 outer electrons 3*s*^2^3*p*^6^3*d*^9^ as the valence electrons), while the all-electron basis set 6-311G** was applied for Se, S, O, N, C and H atoms (all-electron basis set 6-31G** for N, C and H atoms in the neighbouring molecules). Then, natural population analysis was implemented with NBO 3.1 program to estimate the charge distribution in the two temperature structures of **3**. The partial density of states of Cu, O and N atoms in terms of Mulliken population analysis and overlap population density of states between Cu and O/N atoms were also analysed by multiwfn 3.3.8 program (for references on the calculations, see Supplementary Refs 1–12).

### Data availability

The structures have been deposited at the Cambridge Crystallographic Data Centre (deposition numbers: CCDC 1446837–1446845, 1446843 and 1449519), and can be obtained free of charge from the CCDC via www.ccdc.cam.ac.uk/getstructures.

## Additional information

**How to cite this article:** Ye, H.-Y. *et al*. Dielectric and ferroelectric sensing based on molecular recognition in Cu(1,10-phenlothroline)_2_SeO_4_·(diol) systems. *Nat. Commun.* **8,** 14551 doi: 10.1038/ncomms14551 (2017).

**Publisher's note:** Springer Nature remains neutral with regard to jurisdictional claims in published maps and institutional affiliations.

## Supplementary Material

Supplementary Information Supplementary Figures, Supplementary Tables and Supplementary References

Supplementary Data 1 The cif file for compound 1 at 243 K.

Supplementary Data 2 The cif file for compound 1 at 303 K.

Supplementary Data 3 The cif file for compound 1 at 353 K.

Supplementary Data 4 The cif file for compound 2 at 93 K.

Supplementary Data 5 The cif file for compound 2 at 173 K.

Supplementary Data 6 The cif file for compound 2 at 243 K.

Supplementary Data 7 The cif file for compound 2 at 253 K.

Supplementary Data 8 The cif file for compound 2 at 298 K.

Supplementary Data 9 The cif file for compound 2 at 333 K.

Supplementary Data 10 The cif file for compound 3 at 173 K.

Supplementary Data 11 The cif file for compound 3 at 293 K.

Peer Review File
