# Supplementary material for: Dielectric and ferroelectric sensing based on molecular recognition in Cu(1,10-phenlothroline)2SeO4·(diol) systems
Source: Nat Commun. 2017 Feb 20;8:14551. doi: 10.1038/ncomms14551 (PMC5321740; doi:10.1038/ncomms14551)
Supplement: Supplementary Information — Supplementary Figures, Supplementary Tables and Supplementary References [file ncomms14551-s1.pdf]

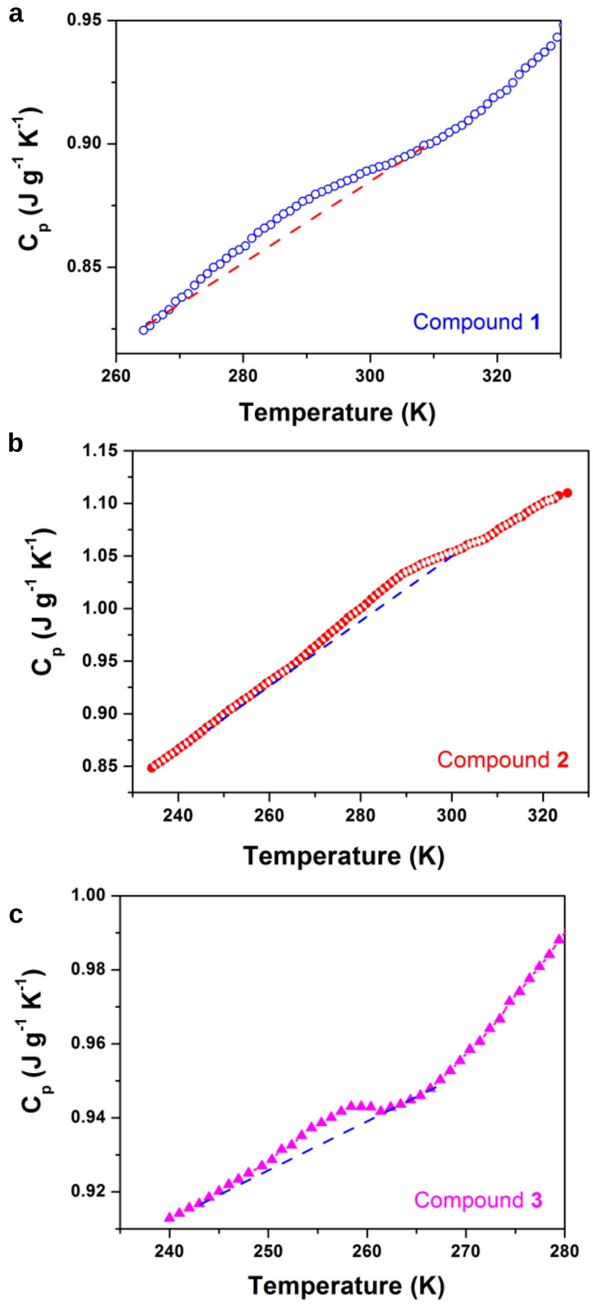

Supplementary Figure 1. Heat capacities of compounds 1–3 as function of temperature. The thermal anomaly indicates a structural phase transition. (**a**) The heat capacity of **1** at various temperature. (**b**) The heat capacity of **2** at various temperature. (**c**) The heat capacity of **3** at various temperature.

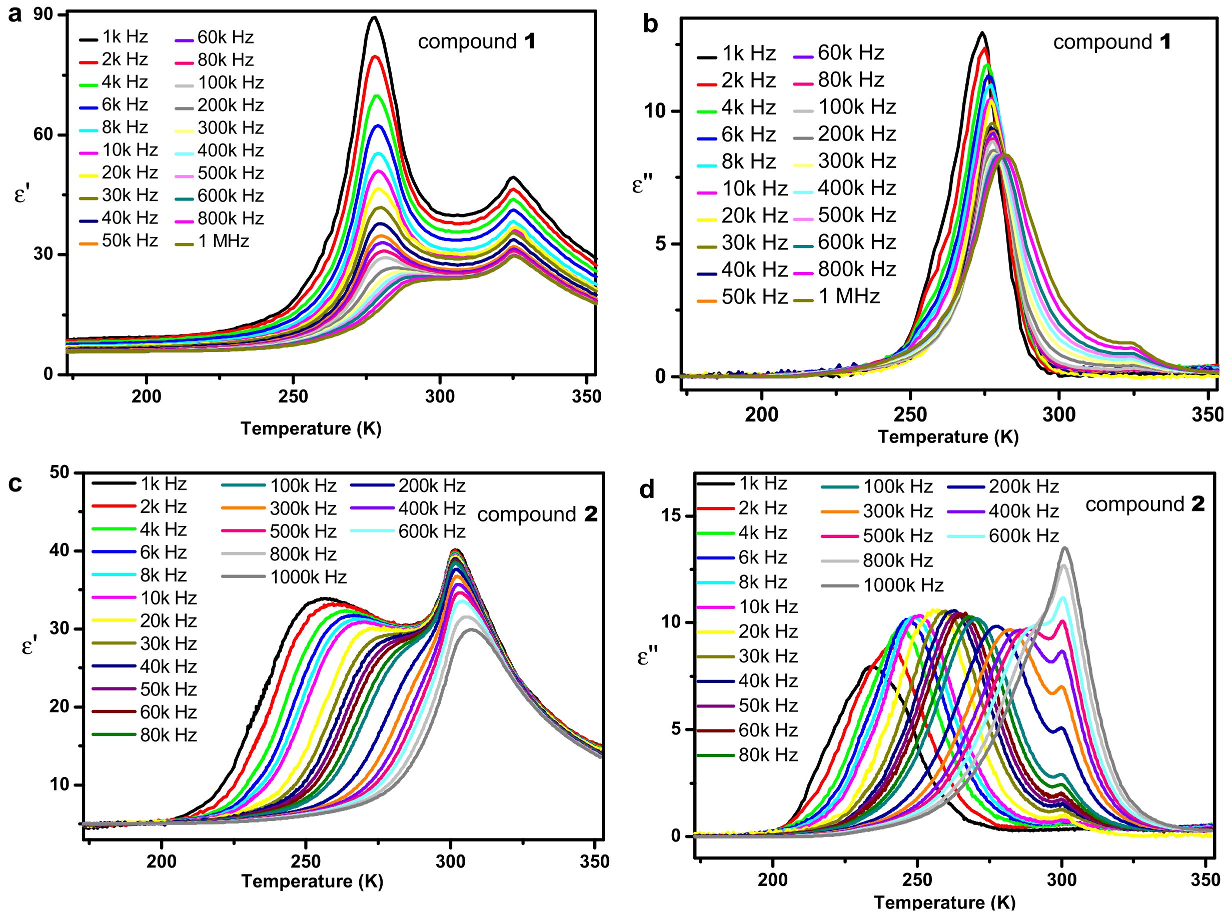

Supplementary Figure 2. Dielectric responses of compounds 1 and 2, showing obvious dielectric dispersion. (**a**, **b**) Temperature dependences of the real part and imaginary part of the complex dielectric constant in compound **1**. **(c**, **d**) Temperature dependences of the real part and imaginary part of the complex dielectric constant in compound **2**.

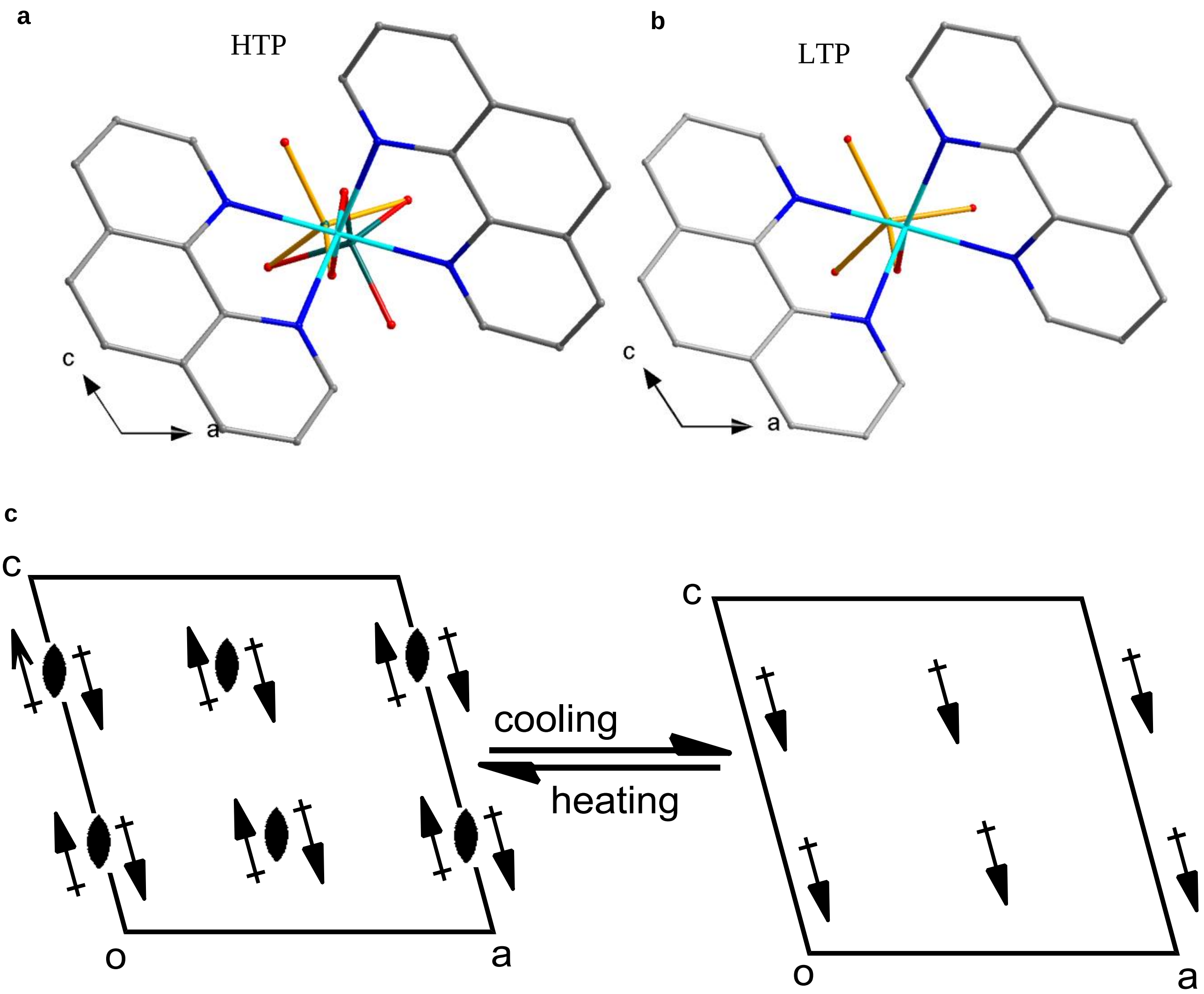

Supplementary Figure 3. Schematic drawing of the polarization mechanism in compound 3. (**a**) The disordered molecule in the high-temperature phase, showing two orientations of the SeO_4^{2-} anion around the crystallographic C_2 axis. (**b**) The ordered molecule in the low-temperature phase, showing the single orientation of the SeO_4^{2-} anion beside the lost C_2 axis. (**c**) Dipole distributions in the *ac* plane in the high and low-temperature phases, showing the polarization mechanism. In **a** and **b**, the propane-1,3-diol molecule and all hydrogen atoms were omitted for clarity. In **c**, only the C_2 axes were shown for clarity.

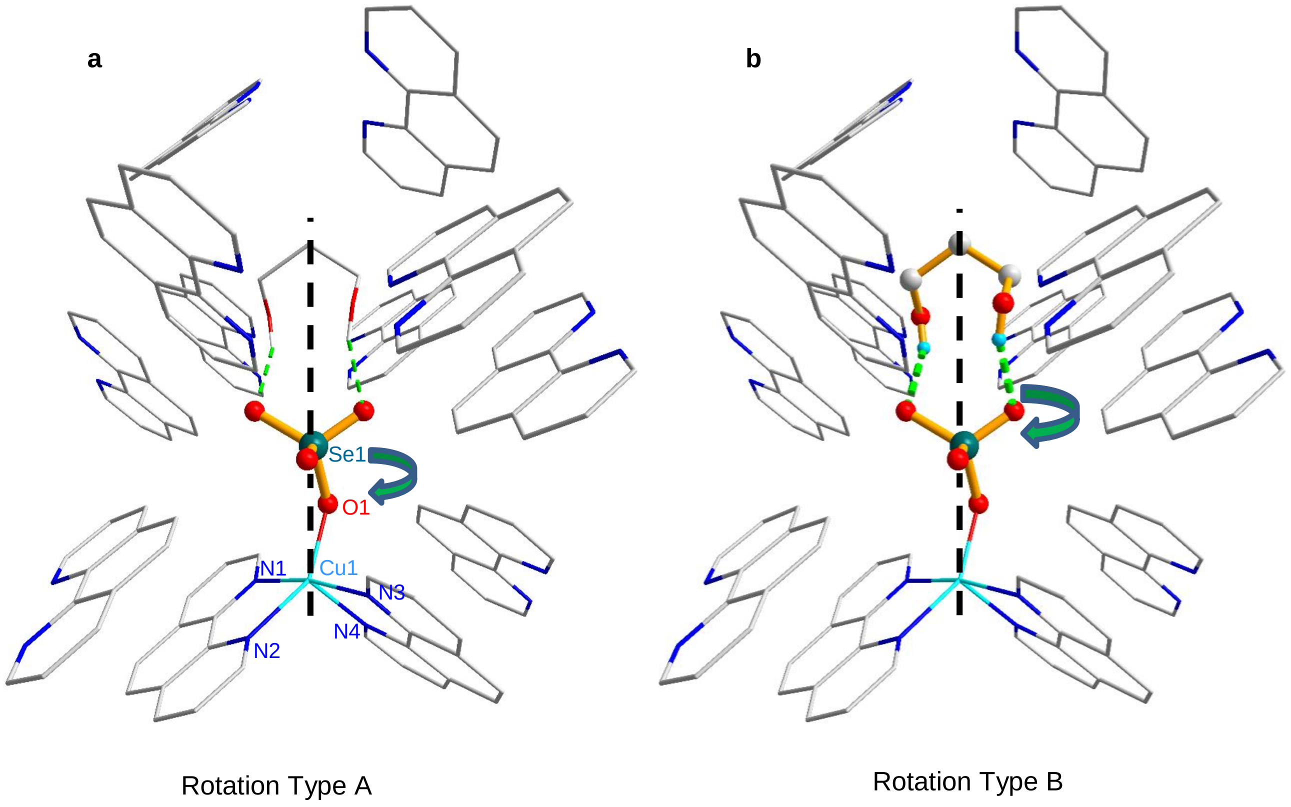

Supplementary Figure 4. Proposed two rotation types. The *C*2/*c* to *Cc* phase transition in compound 3 is realized through one of them. (**a**) Rotation type A, including the rotation of the SeO_4^{2-} anions. (**b**) Rotation type B, including the rotation of the SeO_4^{2-}–propane-1,3-diol as a rigid part. The rotational groups were draw in the ‘ball-and-stick’ model; The molecules bonded to or making weak interaction to the rotational group were draw in the ‘wires/stick’ model. H atoms bonded to the C atom were omitted for clarity.

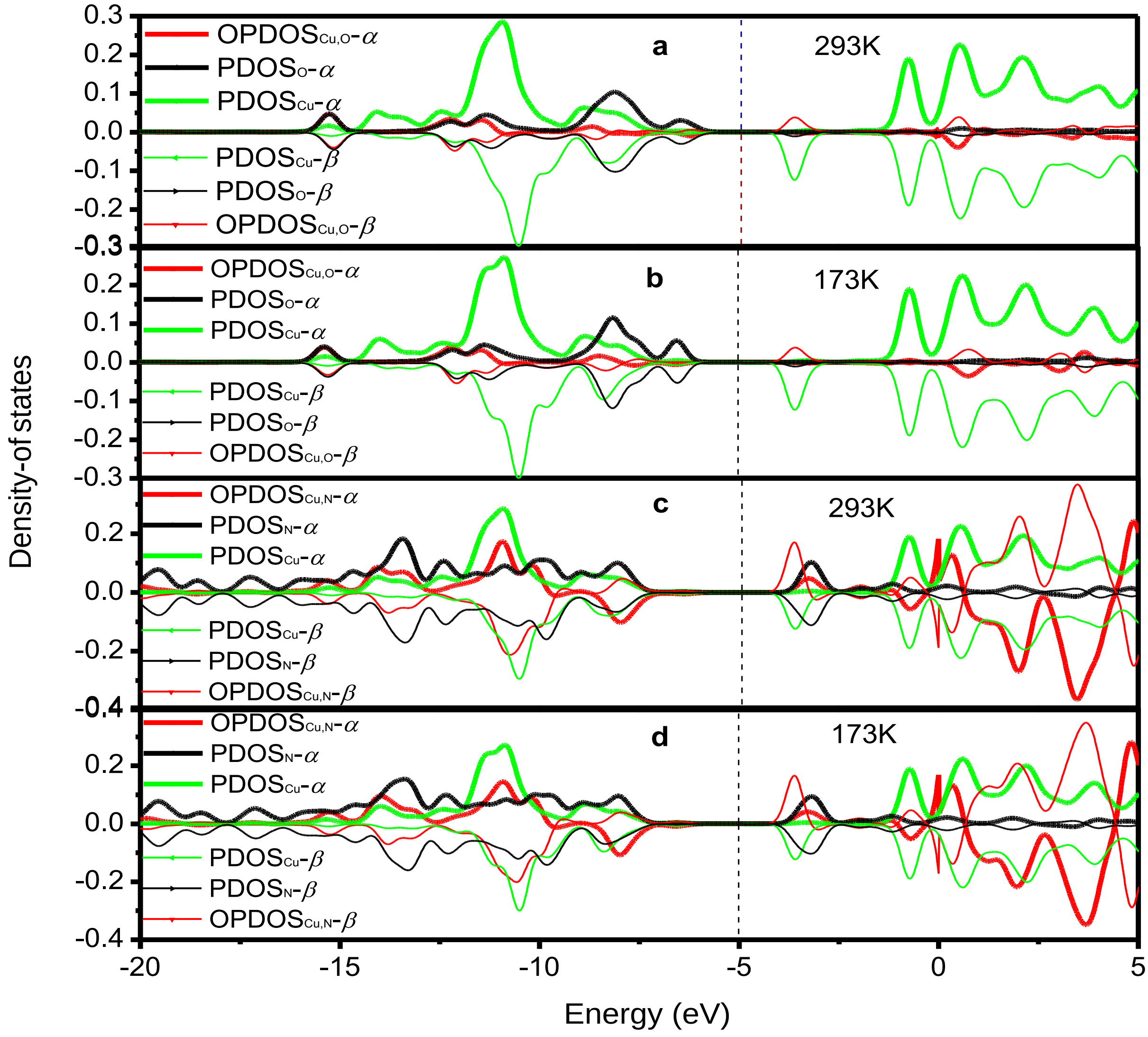

Supplementary Figure 5. Variations of PDOS (partial density-of-states) and the OPDOS (overlap population density-of-states) for compound 3. The results show the similarities of PDOS (partial density-of-states) for Cu, O and N atoms, and the OPDOS (overlap population density-of-states) for Cu-O/N bonds with respect to the energy calculated at B3LYP-D3 level for compound **3** at 293 and 173 K. (**a**, **b**) PDOS of Cu and O atoms and the OPDOS between them at 293K and 173K. (**c**, **d**) PDOS of Cu and N atoms and the OPDOS between them at 293K and 173K. For this open-shell system because of the $3d^9$ electronic structure of Cu^{2+}, the electrons are grouped into two kinds of spin-up (α group) and spin-down (β group). The bold lines represent the α groups, while the thin ones β groups. The dashed lines stand for the position of highest simple occupied molecular orbitals (α/β-SOMO). The values of OPDOS are small in the region below 0 eV, so it is multiplied by 5 to make the variation in the plots clear. The PDOS and OPDOS spectra were produced by convoluting the molecular orbital information with Gaussian broadening function with full width at half maximum (FWHM) of 0.5 eV. In order to plot the PDOS and OPDOS of α/β groups in a whole picture, the PDOS and OPDOS values are multiplied by -1 for the β groups. In the energy region below the level of α/β-SOMO, the values of OPDOS between Cu and O/N atoms are almost positive. So these occupied orbitals are favorable for the formation of Cu-O/N bond.

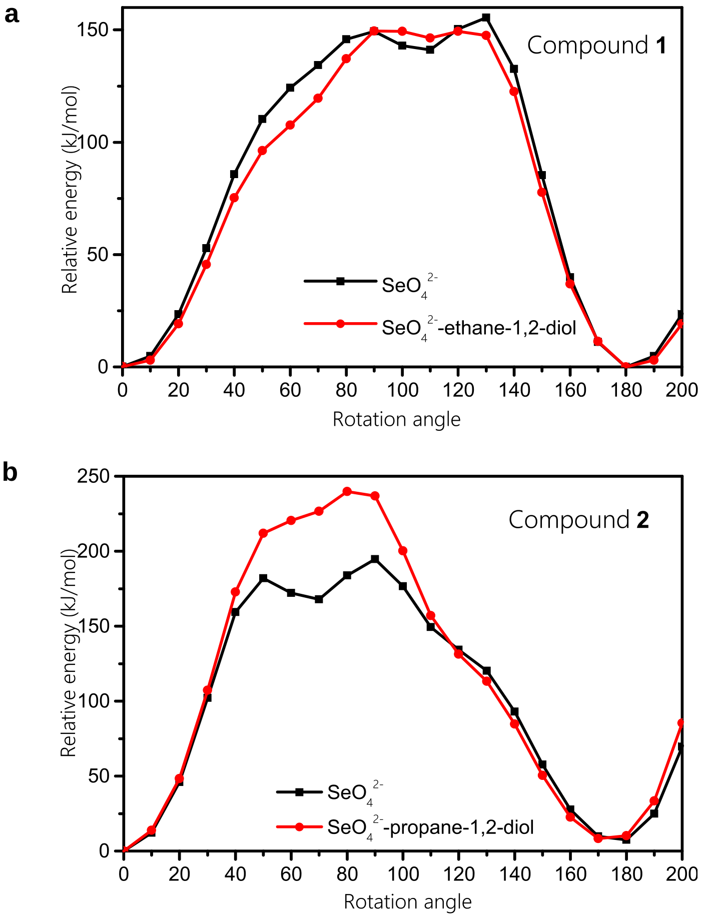

Supplementary Figure 6. Plots of the relative energy for the rotation types A and B in compounds 1 and 2. (**a**) Plots of the relative energy with the rotation angle from 0 °to 200 °for the rotation types A and B in compounds **1**. (**b**) Plots of the relative energy with the rotation angle from 0 °to 200 °for the rotation types A and B in compounds **2**.

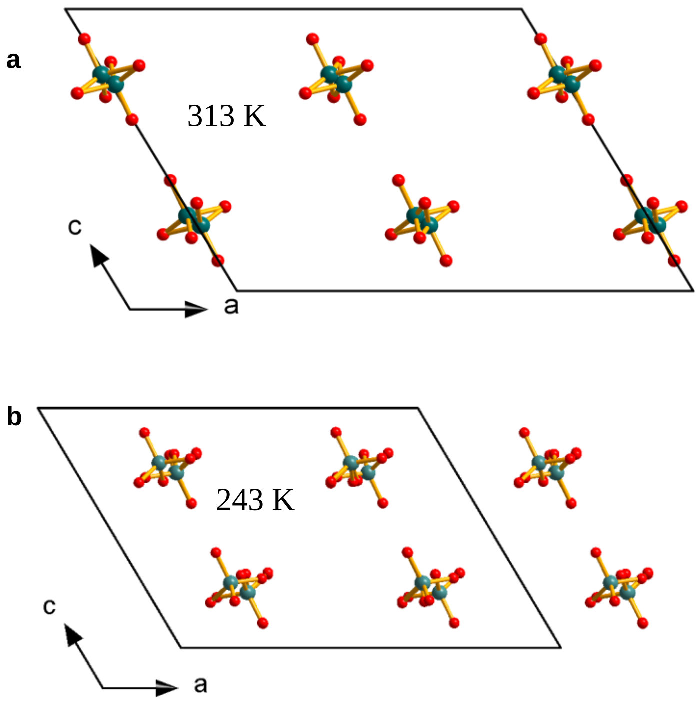

Supplementary Figure 7. Projections along the common *b*-axis of the SeO_4^{2-} anions in compound 1. (**a**) Projections along the common *b*-axis of the SeO_4^{2-} anions of compound **1** in the HTP, showing the equal population of the two orientations in the crystal. (**b**) Projections along the common *b*-axis of the SeO_4^{2-} anions of compound **1** in the LTP, showing the equal population of the two orientations in the crystal.

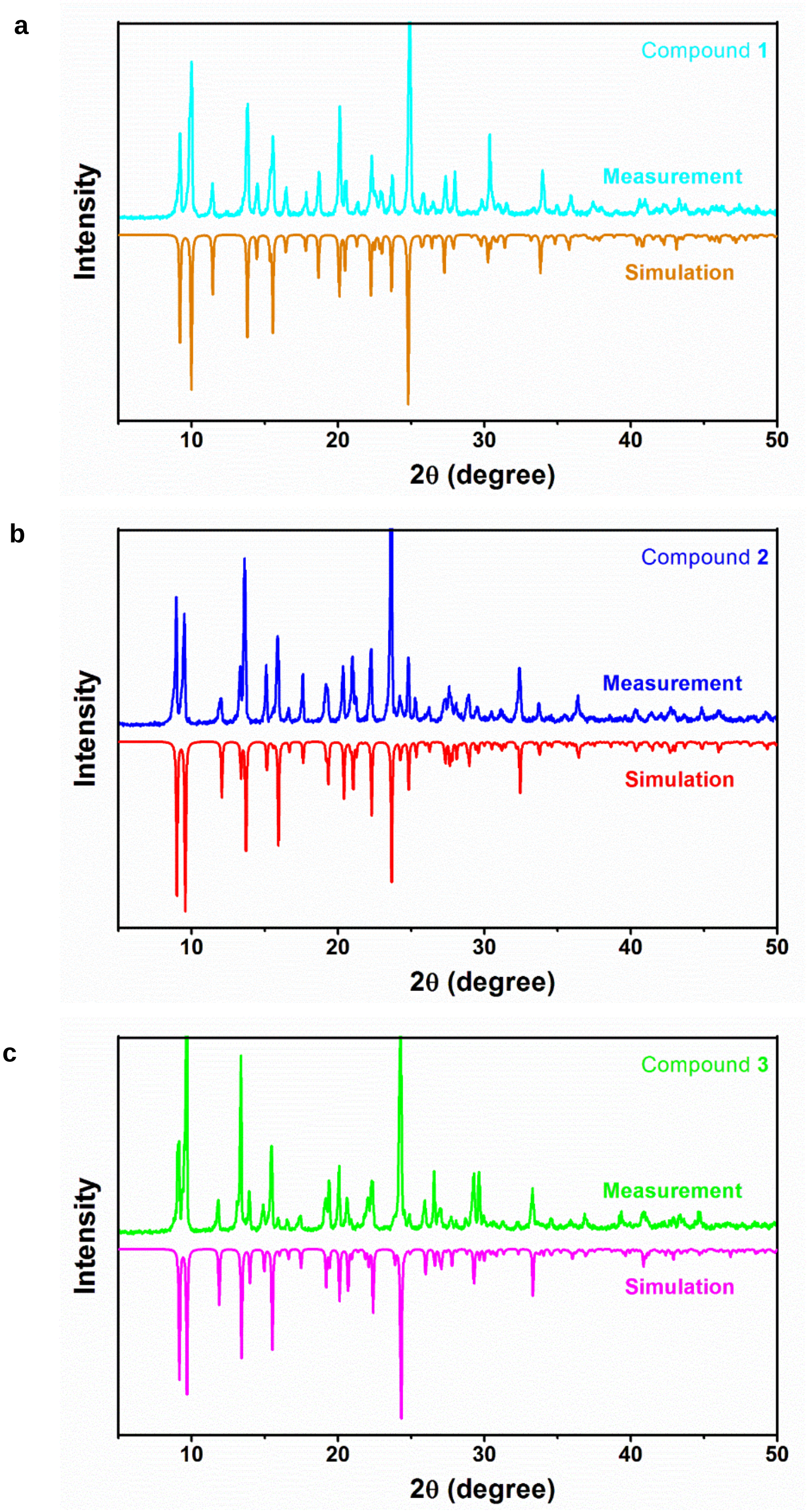

Supplementary Figure 8. Patterns of X-ray powder diffraction. (**a**) The pattern of X-ray powder diffraction for compounds **1**. verifying the purity of the bulk phase. (**b**) The pattern of X-ray powder diffraction for compounds **1**. verifying the purity of the bulk phase. (**c**) The pattern of X-ray powder diffraction for compounds **1**. verifying the purity of the bulk phase.

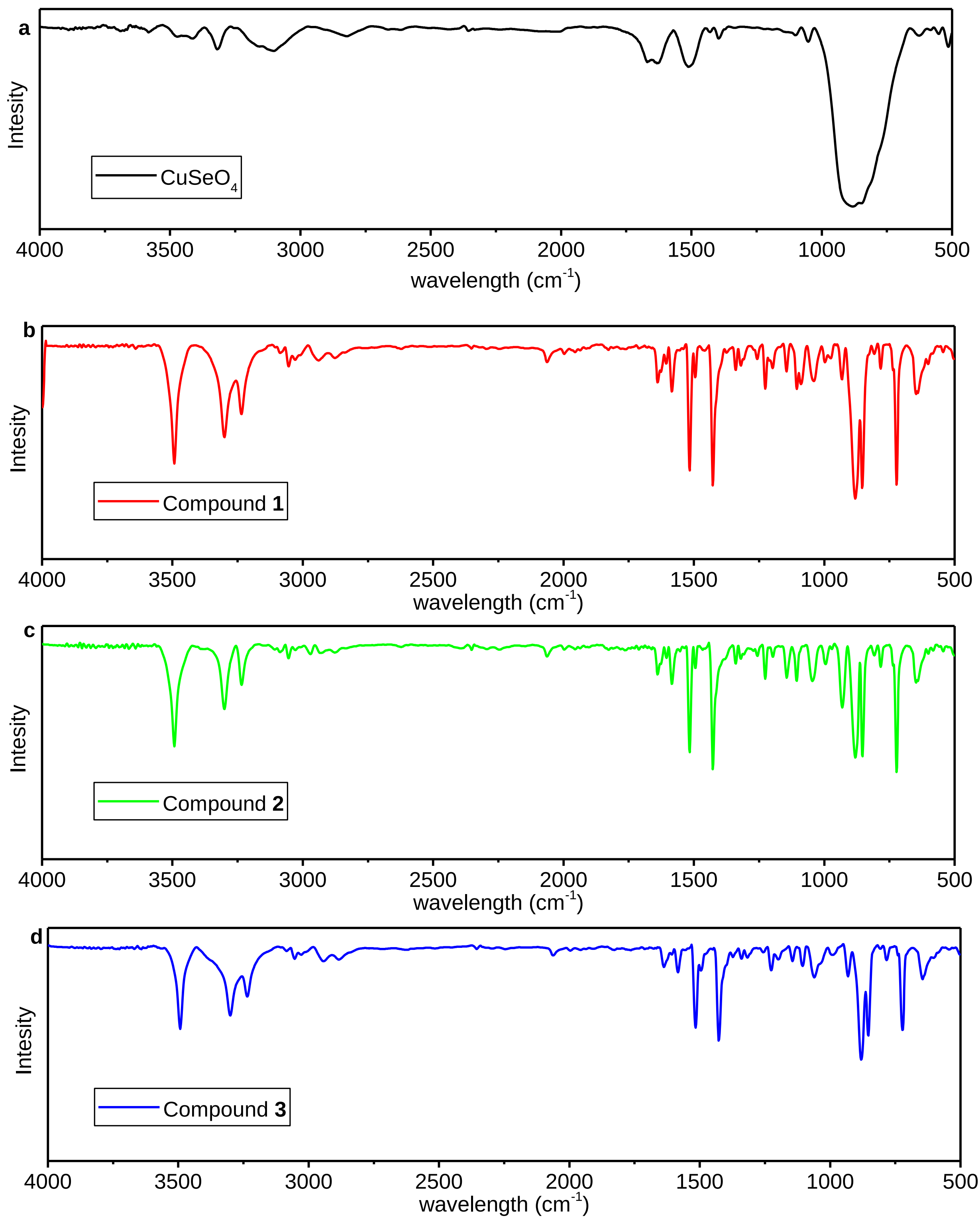

Supplementary Figure 9. Comparison of infrared spectra of 1–3 with that of $CuSeO_4$. The strong absorption at around 877 cm^{-1} in **1**–**3** reveals the existence of SeO_4^{2-} anion. (**a**) The infrared spectrum of $CuSeO_4$. (**b**) The infrared spectrum of compound **1**. (**c**) The infrared spectrum of compound **2**. (**d**) The infrared spectrum of compound **3**.

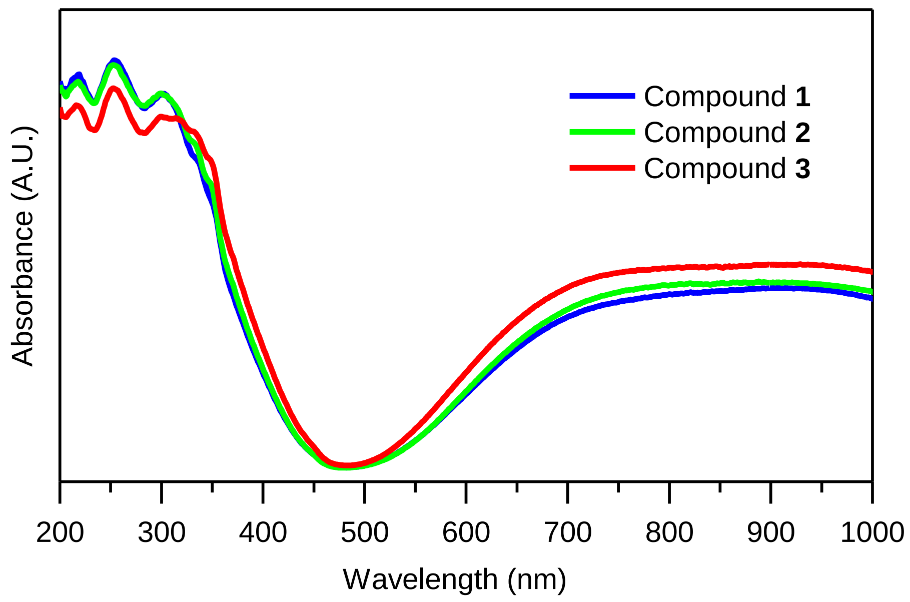

Supplementary Figure 10. UV-vis spectra for 1–3. The absorption at around 480 nm is attributable to the *d*-*d* electron transition of the Cu atom.

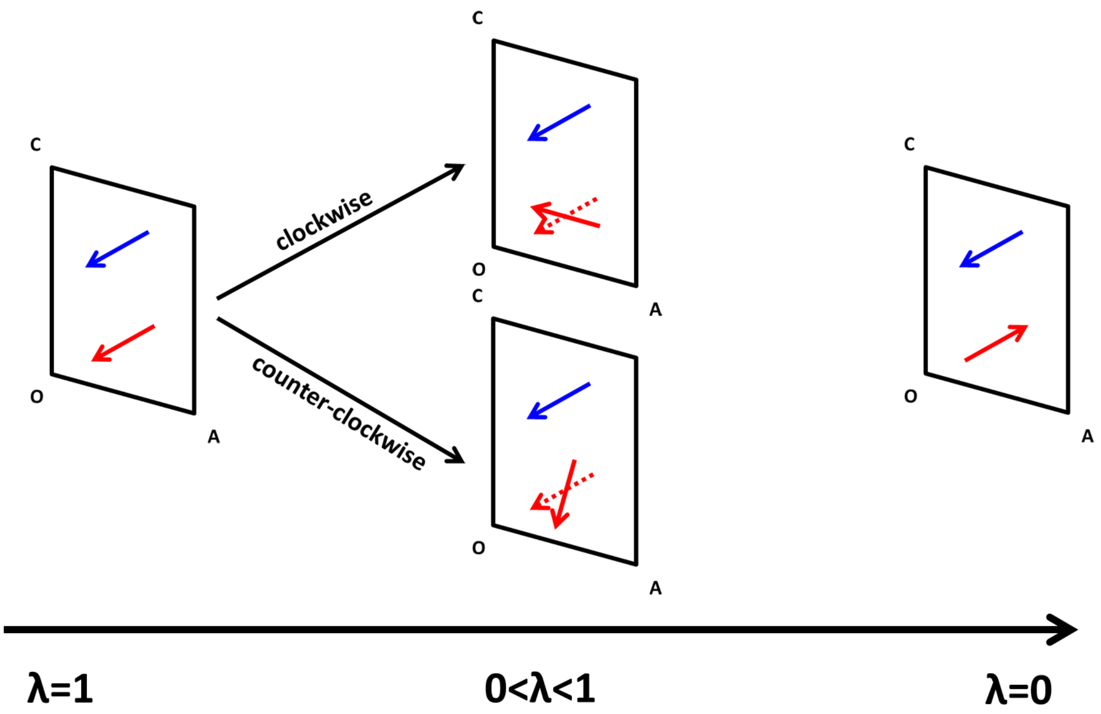

Supplementary Figure 11. Schematic drawing of dipole moment distribution for the polarization calculation.

Supplementary Table 1. Crystal data for compounds 1.

Compound	**1**		
Temperature	353 K	303 K	243 K
Formula, *M*r	$(C_{12}H_8N_2)_2CuSeO_4•C_2H_6O_2$ 628.99		
Crystal system, space group	monoclinic, *C*2/*c*	monoclinic, *C*2/*c*	monoclinic, *P*2_1/*c*
a, b, c (Å)	17.924(13) 12.236(13) 13.253(15)	17.86(3) 12.16(3) 13.17(3)	17.934(12) 36.37(2) 13.179(8)
β (º)	120.69(3)	120.79(3)	120.82(3)
V (Å^3)	2499(4)	2458(9)	7382(7)
*D*c (g cm^{-3})	1.671	1.700	1.698
μ (mm^{-1})	2.381	2.421	2.418
R_1 (I > 2σ(I))	0.0516	0.0697	0.0894
wR_2 (all data)	0.1379	0.1735	0.2324
S	1.202	1.059	1.200

Supplementary Table 2. Crystal data for compounds 2.

Compound	**2**					
Temperature	333 K	298 K	253 K	243 K	173 K	93 K
Formula, *M*r	$(C_{12}H_8N_2)_2CuSeO_4•C_3H_8O_2$ 643.00					
Crystal system, space group	monoclinic, *Cc*	monoclinic, *Cc*	monoclinic, *Cc*	monoclinic, *Cc*	monoclinic, *C*2/*c*	monoclinic, *Cc*
a, b, c (Å)	17.582(8) 13.248(8) 13.324(5)	17.551(6) 13.239(6) 13.305(5)	17.551(18) 13.247(11) 13.266(13)	17.52(2) 13.237(12) 13.262(15)	17.580(19) 13.218(13) 13.269(15)	17.434(19) 13.149(12) 13.184(14)
β (º)	123.38(4)	123.30(3)	123.367(12)	123.415(13)	123.340(13)	123.634(10)
V (Å^3)	2592(2)	2583.8(19)	2576(4)	2576(5)	2567(5)	2516(4)
*D*c (g cm^{-3})	1.648	1.653	1.658	1.658	1.664	1.697
μ (mm^{-1})	2.298	2.305	2.312	2.312	2.320	2.367
R_1 (I > 2σ(I))	0.0391	0.0465	0.0500	0.0509	0.0536	0.0392
wR_2 (all data)	0.0955	0.1264	0.1377	0.1337	0.1438	0.1078
S	1.046	1.025	1.050	1.067	1.004	1.101

Supplementary Table 3. Crystal data for compounds 3.

Compound	**3**	
Temperature	293 K	173 K
Formula,<br>*M*r	$(C_{12}H_8N_2)_2CuSeO_4{\bullet}C_3H_8O_2$<br>643.00	
Crystal system,<br>space group	monoclinic,<br>*C*2/*c*	monoclinic,<br>*Cc*
a, b, c (Å)	17.794(8)<br>12.662(8)<br>13.644(11)	17.723(9)<br>12.711(6)<br>13.471(7)
β (º)	123.19(2)	123.186(8)
V (Å^3)	2573(3)	2540(2)
Dc (g cm^{-3})	1.660	1.682
μ (mm^{-1})	2.315	2.345
R_1 (I > 2σ(I))	0.0411	0.0434
wR_2 (all data)	0.0969	0.0936
S	1.115	1.051

Supplementary Table S4. Fitted Curie constants at different frequencies for compound 3.

Frequence	C_{para} (K)	C_{ferro} (K)	C_{para}/C_{ferro}	T_0 (K)
1 MHz	320	126	2.54	258.1
100 kHz	322	154	2.09	259.3
10 kHz	329	269	1.22	259.7
1 kHz	338	444	0.76	260.2

Supplementary Table 5. The calculated NPA charge distribution for compound 3 at 293K and 173K. The numerical difference between them is smaller than 0.02, indicating that the charge distribution in compound 3 at 293 and 173 K is similar.

Atoms and molecules	293 K	173 K
Cu1	0.992	1.007
Se1	2.737	2.713
O1	-1.063	-1.067
N1	-0.481	-0.487
N2	-0.489	-0.499
N3	-0.489	-0.496
N4	-0.493	-0.494
SeO_4^{2+}-propane-1,3-diol	-1.611	-1.610
1,10-phenanthroline	0.647	0.638
Envir. molecules	-0.028	-0.035

Supplementary References:

1. Kresse, G. & Furthmuller, J. Efficient iterative schemes for ab initio total-energy calculations using a plane-wave basis set. *Phys. Rev. B* **54**, 11169-11186 (1996).
2. Kresse, G. & Furthmuller, J. Efficiency of ab-initio total energy calculations for metals and semiconductors using a plane-wave basis set. *Comput. Mater. Sci.* **6**, 15-50 (1996).
3. Perdew, J. P., Burke, K. & Ernzerhof, M. Generalized gradient approximation made simple. *Phys. Rev. Lett.* **77**, 3865-3868 (1996).
4. Kingsmith, R. D. & Vanderbilt, D. Theory of polarization of crystalline solids. *Phys. Rev. B* **47**, 1651-1654 (1993).
5. Vanderbilt, D. & Kingsmith, R. D. Electric polarization as a bulk quantity and its relation to surface-charge. *Phys. Rev. B* **48**, 4442-4455 (1993).
6. Frisch, M. J. *et al*. Gaussian 09, Revision A.
7. Becke, A. D. Density-functional thermochemistry. III. the role of exact exchange. *J. Chem. Phys.* **98**, 5648-5652 (1993).

8. Lee, C. T., Yang, W. T. & Parr, R. G. Development of the colle-salvetti correlation-energy formula into a functional of the electron-density. *Phys. Rev. B* **37**, 785-789 (1988).

9. Grimme, S., Antony, J., Ehrlich, S. & Krieg, H. A consistent and accurate ab initio parametrization of density functional dispersion correction (DFT-D) for the 94 elements H-Pu. *J. Chem. Phys.* **132**, 154104 (2010).

10. Dolg, M., Wedig, U., Stoll, H. & Preuss, H. Energy-adjusted abinitio pseudopotentials for the first row transition elements. *J. Chem. Phys.* **86**, 866-872 (1987).

11. Glendening, E. D., Reed, A. E., Carpenter, J. E.; Weinhold, F. NBO. Version 3.1.

12. Lu, T. & Chen, F. W. Multiwfn: A multifunctional wavefunction analyzer. *J. Comput. Chem.* **33**, 580-592 (2012).
