# Supplementary material for: Dielectric and ferroelectric sensing based on molecular recognition in Cu(1,10-phenlothroline)2SeO4·(diol) systems
Source: Nat Commun. 2017 Feb 20;8:14551. doi: 10.1038/ncomms14551 (PMC5321740; doi:10.1038/ncomms14551)
Supplement: Peer Review File [file ncomms14551-s13.pdf]

Editorial Note: this manuscript has been previously reviewed at another journal that is not operating a transparent peer review scheme. This document only contains reviewer comments and rebuttal letters for versions considered at Nature Communications.
